# Spatial Assessment of Heterogeneous Tissue Natural Frequency Using Micro-Force Optical Coherence Elastography

**DOI:** 10.3389/fbioe.2022.851094

**Published:** 2022-03-11

**Authors:** Gongpu Lan, Qun Shi, Yicheng Wang, Guoqin Ma, Jing Cai, Jinping Feng, Yanping Huang, Boyu Gu, Lin An, Jingjiang Xu, Jia Qin, Michael D. Twa

**Affiliations:** ^1^ School of Physics and Optoelectronic Engineering, Foshan University, Foshan, China; ^2^ Innovation and Entrepreneurship Teams of Guangdong Pearl River Talents Program, Weiren Meditech Co., Ltd., Foshan, China; ^3^ Guangdong-Hong Kong-Macao Intelligent Micro-Nano Optoelectronic Technology Joint Laboratory, Foshan University, Foshan, China; ^4^ School of Mechatronic Engineering and Automation, Foshan University, Foshan, China; ^5^ Institute of Engineering and Technology, Hubei University of Science and Technology, Xianning, China; ^6^ School of Computer and Information Engineering, Tianjin Chengjian University, Tianjin, China; ^7^ College of Optometry, University of Houston, Houston, TX, United States

**Keywords:** optical coherence tomography, optical coherence elastography, natural frequency, soft-tissue biomechanics, ophthalmology

## Abstract

Analysis of corneal tissue natural frequency was recently proposed as a biomarker for corneal biomechanics and has been performed using high-resolution optical coherence tomography (OCT)-based elastography (OCE). However, it remains unknown whether natural frequency analysis can resolve local variations in tissue structure. We measured heterogeneous samples to evaluate the correspondence between natural frequency distributions and regional structural variations. Sub-micrometer sample oscillations were induced point-wise by microliter air pulses (60–85 Pa, 3 ms) and detected correspondingly at each point using a 1,300 nm spectral domain common path OCT system with 0.44 nm phase detection sensitivity. The resulting oscillation frequency features were analyzed via fast Fourier transform and natural frequency was characterized using a single degree of freedom (SDOF) model. Oscillation features at each measurement point showed a complex frequency response with multiple frequency components that corresponded with global structural features; while the variation of frequency magnitude at each location reflected the local sample features. Silicone blocks (255.1 ± 11.0 Hz and 249.0 ± 4.6 Hz) embedded in an agar base (355.6 ± 0.8 Hz and 361.3 ± 5.5 Hz) were clearly distinguishable by natural frequency. In a beef shank sample, central fat and connective tissues had lower natural frequencies (91.7 ± 58.2 Hz) than muscle tissue (left side: 252.6 ± 52.3 Hz; right side: 161.5 ± 35.8 Hz). As a first step, we have shown the possibility of natural frequency OCE methods to characterize global and local features of heterogeneous samples. This method can provide additional information on corneal properties, complementary to current clinical biomechanical assessments, and could become a useful tool for clinical detection of ocular disease and evaluation of medical or surgical treatment outcomes.

## Introduction

Soft tissue biomechanics (e.g., stiffness, elasticity, and viscosity) are highly dependent upon tissue structure, materials, and composition, and they often change alongside pathological changes, such as swelling, inflammation, and tumor growth (Dupps and Wilson, 2006; Sigrist et al., 2017; Chong and Dupps, 2021). Elastic imaging methods, such as ultrasound elastography (Ophir et al., 1991) and magnetic resonance imaging (MRI) elastography (Muthupillai et al., 1995), have been developed to identify lesion areas based on mechanical contrast (e.g., strain map or wave propagations). These methods have been widely used in the diagnosis of diseases such as liver cirrhosis, breast fibrosis, and cancer (Sigrist et al., 2017).

In ophthalmology, corneal elasticity assessment is essential in ocular disease detection and management, such as diagnosing and classifying keratoconus progression (Shah et al., 2007; Roberts and Dupps, 2014; Scarcelli et al., 2014; Bao et al., 2016; Alvani et al., 2020; Tian et al., 2021), preoperatively screening refractive-surgery candidates who are at higher risk of postoperative ectasia (Kanellopoulos, 2007; Shetty et al., 2017; Dackowski et al., 2020; Salomão et al., 2020; Song et al., 2021), and evaluating medical or surgical treatment outcomes (Seven et al., 2016; Bao et al., 2018; Ferguson et al., 2021). Ocular Response Analyzer (ORA, Reichert Inc. Buffalo, NY) (Luce, 2005) and CorVis ST (OCULUS, Inc. Arlington, WA) (Hon and Lam, 2013) are two clinically available devices for assessing intraocular pressure and corneal biomechanics. These two clinical devices are based on an inward corneal applanation event generated by air-puff and an outward applanation event as the cornea recovers to its original shape. Both have been proven reliable in intraocular pressure measurement, but they are still limited in corneal biomechanics evaluation because the estimated corneal biomechanical properties are generally global properties rather than local properties, which would reflect subtle changes in the cornea. Large-amplitude (e.g., 70–300 kPa) stimulation force can result in global corneal deformation, ocular motion, aqueous fluid displacement, as well as globe retraction and rotation (Boszczyk et al., 2017; Jiménez-villar et al., 2019). These factors confound the measurements of these ocular biomechanics measurement methods and limit the capability for detecting regional variations in corneal stiffness (Singh et al., 2017). Previous clinical studies have shown conflicting results: although corneal biomechanics could be differentiated between normal and keratoconus eyes by ORA (Gkika et al., 2012) and CorVis ST (Bak-Nielsen et al., 2014), no significant differences have been found in keratoconus eyes before and after cross-linking treatments (Gkika et al., 2012; Greenstein et al., 2012; Bak-Nielsen et al., 2014). To date, measuring corneal biomechanical properties *in vivo* remains challenging, and there is no gold standard for assessing corneal biomechanics spatially and locally.

Optical coherence elastography (OCE) was developed by combining a loading system to exert a sample stimulation force and an optical coherence tomography (OCT) system to observe the resulting tissue displacements (strains) or mechanical waves (Schmitt, 1998). OCT/OCE provides micrometer-scale axial and lateral resolutions, and the use of phase-sensitive OCT detection (Zhao et al., 2000; Kirkpatrick et al., 2006) can further enhance the dynamic elastography detection sensitivity to a sub-nanometer scale (Lan et al., 2017), making it possible to detect minute-magnitude dynamics for *in vivo* human corneas (Lan et al., 2020a; Ramier et al., 2020; Lan et al., 2021b). The measurement of tissue biomechanics typically centers on Young’s modulus, a representation of elasticity expressed as the slope between the force (stress) and the resulting fractional deformation (strain). By analogy with ultrasound elastography, the most commonly used OCE method is based on the measurement of shear (or surface) elastic-wave propagation velocities for Young’s modulus estimation (Doyle, 1997; Song et al., 2013a; Wang and Larin, 2014; Zvietcovich and Larin, 2021). However, it has been frequently reported that the shear wave model is useful in bulk tissues/organs, such as the liver and kidney (using ultrasound- or MRI-based elastography detection), but would likely fail in tissues with thin layers and complex boundary conditions, such as the cornea and skin, hence the interest in developing alternative methods (Pelivanov et al., 2019). In the cornea, propagating mechanical waves are not ideal simple Rayleigh waves. Instead, they may contain many highly dispersive Rayleigh-Lamb components requiring a more complex analytic model for accurate interpretation (Han et al., 2015a; Pitre Jr et al., 2019). Thus, the use of a simple shear wave model could have large errors for Young’s modulus estimation (Pelivanov et al., 2019). Although a modified Rayleigh-Lamb wave model was presented for corneal viscoelasticity assessment (Han et al., 2015a; Han et al., 2017), this method is based on a first-order assumption that the cornea is isotropic, homogenous, and has a flat curvature. Developing more robust computational methods and tissue modeling techniques is important for enhancing tissue elasticity estimation accuracy in OCE applications (Larin and Sampson, 2017).

In addition to strain- and wave-based OCE strategies, resonant OCE approaches have been developed to quantify sample natural frequencies (the frequencies at which sample tends to oscillate when disturbed) based on the vibrational or resonant response of samples under harmonic or chirp stimulation forces. A variety of modulating forces have been used in resonant OCE approaches, such as acoustic radiation force from ultrasound transducers (Qi et al., 2013), piezoelectric actuators (Adie et al., 2010) or mechanical wave drivers (Liang et al., 2008), magnetic force from embedded nanoparticle transducers (Crecea et al., 2009; Oldenburg and Boppart, 2010; Fischer, 2011), and audio sound waves from a speaker (Akca et al., 2015). Previous studies have shown frequency-enhanced mechanical contrast in cross-sectional or volumetric imaging for *ex vivo* samples (Liang et al., 2008; Adie et al., 2010; Fischer, 2011; Qi et al., 2013), high-resolution measurement of resonant natural frequencies in a stimulation frequency range (Crecea et al., 2009; Adie et al., 2010; Oldenburg and Boppart, 2010; Fischer, 2011; Qi et al., 2013), and the linear relation between the natural frequency and the square root of Young’s modulus in a simple elastic model (Crecea et al., 2009; Qi et al., 2013).

These stimulation methods are not suitable for *in vivo* corneal measurements. The labelling agent methods (Crecea et al., 2009; Oldenburg and Boppart, 2010; Fischer, 2011) are not safe, and the mechanical contact (Liang et al., 2008; Adie et al., 2010) methods may be unsuitable for *in vivo* ocular measurements. Although audio frequency-based OCE was implemented to observe response frequencies from bovine eyes *ex vivo* (Akca et al., 2015); the induced large-scale tissue vibrations (in millimeter scale) require large stimulation forces that are potentially hazardous for ocular tissues *in vivo* as well. In addition, the frequency sweeping method (i.e., using swept harmonic signals over a defined frequency range, or chip signals) usually takes longer time, and could cause discomfort or harm during *in vivo* measurements for the human eye. Transient tissue stimulation methods (e.g., impulse stimulation functions (Wang et al., 2013) and square-wave modulation (Crecea et al., 2009)) can provide broadband stimulation frequencies simultaneously, thereby reducing acquisition time, safety, and comfort for patients. Conversely, wider temporal stimulus duration results in narrower frequency bandwidth responses (Ramier et al., 2019; Zvietcovich and Larin, 2021).

In our previous work, we proposed an OCE method for natural frequency quantification using a microliter air-pulse stimulator to provide transient (∼1–4 ms) and broadband frequency excitation (e.g., ∼0–1 kHz for a 1-ms duration pulse) with induced tissue damping oscillation magnitudes in sub-micrometer- to sub-nanometer range. Subsequently, we used the method for *in vivo* corneal biomechanics assessment. The dominant tissue oscillation features, such as the dominant natural frequency, decay coefficient, and damping ratio, can be analyzed by utilizing a single degree of freedom (SDOF) quantification method (Lan et al., 2020b). Compared to the *in vivo* OCE measurements of corneal displacements (average coefficient of variation: 17.0%, magnitude: 0.2–0.8 μm (Lan et al., 2020a)) or corneal surface wave speeds (average coefficient of variation: 19.3%, 2.4–4.2 m/s for 18 eyes (Lan et al., 2021b)), the *in vivo* OCE measurements of corneal natural frequency have much better repeatability and reproducibility (average coefficient of variation: 3.2%, 234–277 Hz for 20 eyes (Lan et al., 2021a)). This natural frequency OCE approach could be complementary to the current OCE methods used to estimate Young’s modulus from strain- or shear-wave-based measurements for the quantitative determination of corneal biomechanics.

However, there are questions remaining to be solved before we can apply this natural frequency OCE method in eye clinics. Previous studies have focused on characterizing global tissue properties of phantoms and shown the dependency of natural frequencies on the mass, thickness, and stiffness of whole phantoms (Lan et al., 2020b). However, previous studies have not provided any proof as to whether the measurement of natural frequency can be used to distinguish local variations in heterogeneous tissues or samples, such as variations in tissue sub-organizations, interfaces between adjacent tissue components or materials, or stiffness due to injury or pathological progression. Although we have noticed spatial variations of natural frequency in the *in vivo* measurement of the cornea (Lan et al., 2021a), we do not know what parameters caused the natural frequency variations, how they caused the variations, and to what degree each parameter contributed to the variations. Therefore, the goal of this study was to discover the possibility and application scope of the natural frequency OCE method for global and local characterization of corneal biomechanics in a clinical setting. Interpretation of the corneal natural frequency response is not straightforward because the cornea has a very complex microstructure, is spatially inhomogeneous in the lateral and depth directions, and is directionally anisotropic in its response to load according to its structural subcomponents and organizations and its hydration state (Dupps and Wilson, 2006; Meek, 2009; Chong and Dupps, 2021). In this study, as a first step, we focused on the spatial characterization of the oscillation frequency features and the natural frequencies in heterogeneous samples much simpler than the cornea—specifically, agar-silicone phantoms and beef tissue samples. Development of these analytical methods may enable future applications for the use of natural frequency to characterize local tissue properties including corneal elastography and biomechanics estimation *ex vivo* and *in vivo*.

## Materials and Methods

### Samples

Agar and silicone phantoms are commonly used as corneal-mimicking phantoms (Li et al., 2013; Han et al., 2015b; Wang et al., 2018). In this study, we used agar/silicone tissue-mimicking phantoms and beef tissue samples to assess the spatial distributions of natural frequencies. Four types of phantoms were made: a pure silicone phantom (Shore hardness: 25, hereafter referred to as the silicone phantom), a pure 2% agar phantom (hereafter referred to as the agar phantom), and two agar phantoms containing silicone blocks of different shapes (hereafter referred to as silicone-agar mixture phantoms). Each phantom filled a Petri dish with an inner diameter of 57 mm and depth of 14 mm. The use of the Petri dish was to mimic a similar and simplified boundary condition of the cornea by providing an extra constraint in the sample’s circumferential direction. The agar phantom (Biowest agarose 111,860) was made following the procedures described previously (Li et al., 2013; Han et al., 2015b; Lan et al., 2020b). The silicone phantom was prepared by mixing silicone components A and B (Shenzhen Ketai Technology Co., Ltd., Shenzhen, China) in a ratio of 1:1 and pouring the mixture into a Petri dish. After 24 h at room temperature, the phantoms were completely dry and ready to be used for measurement. The densities of silicone and 2% agar were 936 kg/m^3^ and 933 kg/m^3^, respectively. The Young’s moduli of the silicone (757.99 kPa) and agar phantoms (1,061.40 kPa) were measured under a 10 N force using a TH-8203A mechanical test frame (Wane Testing Equipment Co., Ltd, Suzhou, Jiangsu, China). Two silicone-agar mixture phantoms were made from two silicone blocks—a rectangular one (length: 5 mm, width: 3 mm, height: 12 mm) and a triangular one (sides: 4–5.5 mm, height: 12.5 mm)—and then each block was embedded in 2% agar. The top of each silicone block was also sealed with agar to achieve a total height of 14 mm. The phantoms roughly mimic tissue lesions or inclusions surrounded by normal tissues. Beef shank samples were bought from a local market, cut into small pieces, and sealed inside 2% agar in a Petri dish with an inner diameter of 38 mm and inner height of 13 mm. Samples were selected to contain muscle, fat, and connective tissues for natural frequency OCE measurements.

### OCE Setup

**FIGURE 1:**
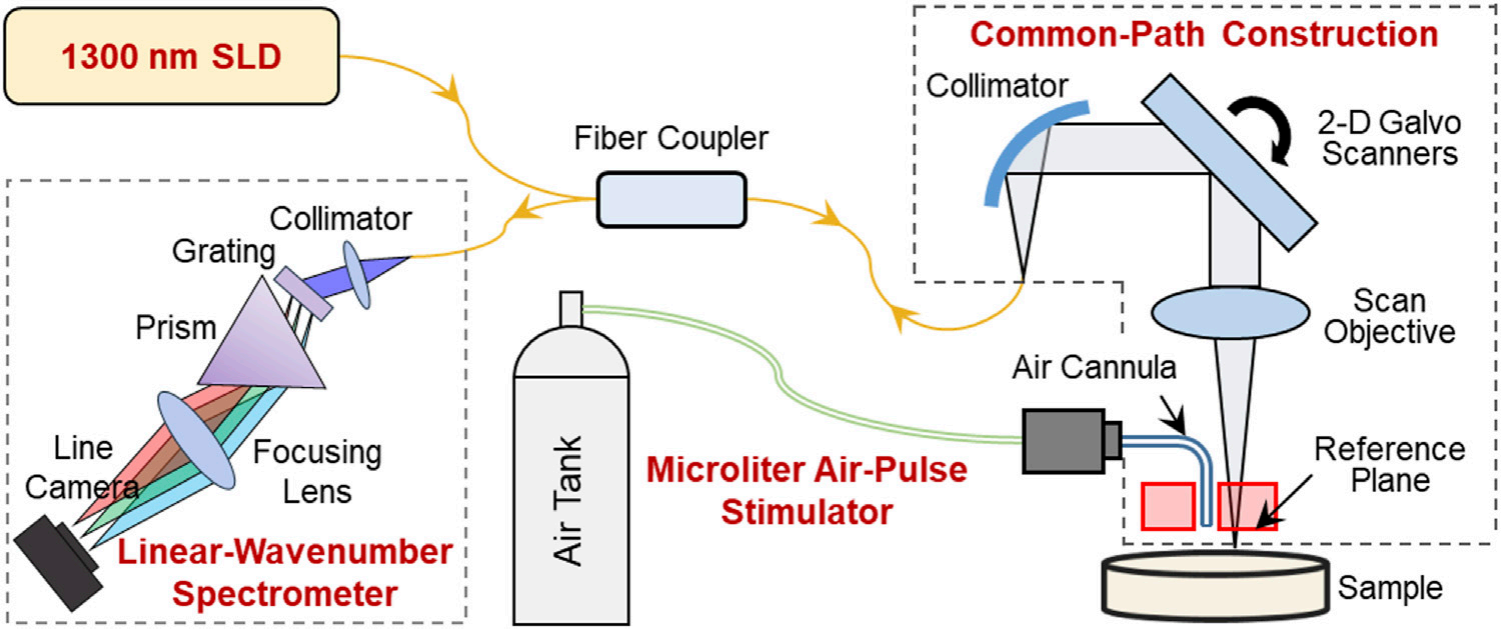
Schematic of the optical coherence elastography (OCE) system. A microliter air pulse stimulator was used to evoke submicron-scale mechanical waves in the sample, and a high-resolution phase-sensitive OCT imaging system was used to track and record the tissue response dynamics. In the OCE imaging subsystem, SLD is a superluminescent laser diode with a waveband of 1,290 ± 40 nm; the sample and reference arms share a common optical path to enhance the phase detection stability; and a linear-wavenumber spectrometer was used to disperse the interference spectrum optically in the wavenumber domain to enhance imaging detection sensitivity.

A home-made OCE system was constructed from a microliter air-pulse mechanical stimulation system and combined with a linear-wavenumber common-path spectral domain OCT platform, as shown in Figure 1. The microliter air-pulse stimulation system was controlled with a high-speed solenoid valve that delivered micro-mechanical stimulation perpendicular to the sample surface through a microbore cannula. The cannula provided a spatially-focused (150 μm in diameter), low-pressure (60–85 Pa), and short duration (approximately 3 ms) stimulation force to the samples. The cannula tip was inserted through a hole created in the common-path OCT system reference plate and mounted flush with the surface. The OCT imaging system was synchronized to the stimulation system to record the dynamic response of the sample. The minimum lateral distance measurable by the OCT system was 0.15 mm from the stimulation point.

The OCT/OCE techniques are described in detail in our previous work (Lan et al., 2017; Lan et al., 2021c). In summary, the OCT system was built upon a 1,300 nm linear-wavenumber spectral domain OCT platform (Lan et al., 2021c). The light emitted from a superluminescent diode (SLD, IPSDS1307C-1311, Inphenix Inc., Livermore, CA, United States) with a 3 dB bandwidth of 1,290 ± 40 nm was split equally into sample and reference arms. The reference arm was blocked, whereas a 4 mm thick acrylic reference plate was used in the sample arm to provide a reference plane (the optical surface proximal to the sample). Hence, the sample and reference arms shared a common optical path (Figure 1). The common-path construction effectively reduced the amplitude of the background phase disturbance caused by environmental vibrations and improved the phase detection sensitivity (Lan et al., 2017). In the common path, the maximum output power was 1.8 mW, and the output light was collimated by a reflective collimator (RC04APC-P01, Thorlabs, Inc., Newton, NJ, United States) with 4 mm beam diameter, scanned by 2D galvo mirrors (GVS102, Thorlabs Inc., Newton, NJ, United States), and focused by a telecentric scan objective (LSM54-1,310, Thorlabs Inc., Newton, NJ, United States) with a focal length of 54 mm and a maximum field of view of 18.8 × 18.8 mm^2^. The interference spectrum generated from the sample and the reference plane was recorded by a linear-wavenumber spectrometer (PSLKS1300-001-GL, Pharostek, Rochester, Minnesota, United States) equipped with an InGaAs line scan camera (GL2048L-10A-ENC-STD-210, Sensors Unlimited, Inc., Princeton, NJ, United States) at a line rate of 76 kHz. The linear-wavenumber spectrometer dispersed the spectrum optically in the wavenumber domain instead of the wavelength domain so that no additional digital interpolation was acquired prior to Fourier transform (Hu and Rollins, 2007; Lan and Li, 2017). Finally, the interference signals were transported through a frame grabber (PCIE-1433, National Instruments Corp., Austin, Texas, United States) into a computer and were then processed directly via Fourier transform to acquire depth profiles (A-scans) using code written in the LabVIEW language. In air, the maximum imaging depth was 6.94 mm for 1,024 pixels (each pixel corresponded to 6.78 µm), the maximum sensitivity was 99.3 dB, the −6 dB fall-off range is ∼0–3 mm deep, the maximum sensitivity fall-off is −28.6 dB, and the axial resolution was ∼15 µm (2 pixels).

In the transformed OCT interferogram signal, the real component (intensity) illustrates the structural imaging, and the phase signal can be generated by analyzing the complex component. Structural imaging is limited to micron-scale axial resolution, while phase-sensitive detection techniques enhance the dynamic elastography detection sensitivity to a sub-nanometer scale. During micro-force OCE measurements, the tissue displacements were much smaller than the axial resolution (∼15 µm in this system) or one pixel (6.78 µm). The tissue surface was tracked using structural imaging, and the sub-pixel tissue surface displacements *y*(*t*) for each measurement point in the time (*t*) domain was calculated from the phase change among successive A-scan signals (Song et al., 2013b):

$$y\left(t\right)=\frac{\lambda_{0}}{4\mathrm{\pi n}}\phi \left(t\right)$$ (1)

where *Φ*(*t*) represents the phase change value after phase-unwrapping processing, *λ*_*0*_ is the center wavelength, and *n* is the refractive index (*n* = 1 in air). In the absence of applied forces, the average phase variation was measured using a mirror as 4.3 ± 1.4 milliradians over 60 ms (10 repetitive measurements) in the common-path OCT setup, which corresponded to displacements of 0.44 ± 0.14 nm according to Eq. 1.

### Oscillation Frequency Spectrum and the Dominant Natural Frequency

**FIGURE 2:**
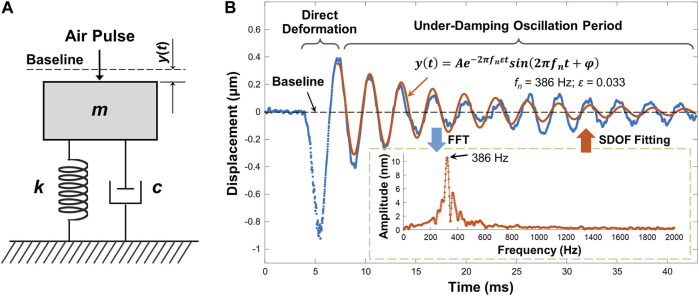
Natural frequency characterization using single degree of freedom (SDOF) model. **(A)** Typical spring-mass-damper system. *m*: mass; *k*: spring stiffness coefficient; *c*: viscous damping coefficient; *y*(*t*): displacement dynamics. **(B)** Typical sample displacement profile and the SDOF fitting for its damping oscillation period (∼7.2–42.9 ms). In this example, the sample was the agar phantom. The embedded figure shows the FFT results for the damping oscillation period. The dominant oscillation frequency was substituted into Eq. 3 to fit the oscillation period.

Tissue natural frequency is an intrinsic property, defined as the frequency at which samples tend to oscillate when disturbed. Natural frequency is determined by factors such as spring stiffness, mass, thickness, size, shape, and boundary conditions, but is not determined by the stimulation force or stimulation frequency. In our previous work (Lan et al., 2020b), the natural frequency concept using OCE measurements was verified on pure agar phantoms: the measured natural frequency was constant for different stimulation pressures and measured distances, and decreased as the sample thickness increased. A single degree of freedom (SDOF) model was used to analyze the dominant natural frequency component with the maximum oscillation magnitude. The SDOF model was built in a simple spring-mass-damper system (Figure 2A) to quantify the sample natural frequency. In the SDOF model, the sample natural frequency *f*_*n*_ can be represented as $f_{n}=\sqrt{k/m}/2\pi$ , where *m* is the mass, and *k* is the spring stiffness coefficient; and the damping ratio *ε* is defined as $\varepsilon =c/\left(4\pi mf_{n}\right)$ , where *c* is the viscous damping coefficient. The response oscillation can be described as three different oscillation regimes: critical-damping (damping ration *ε* = 1), under-damping (0 ≤ *ε* < 1), and over-damping (*ε* > 1).

Figure 2B demonstrates a typical air-pulse generated surface displacement profile from OCE measurements. The air-pulse duration was ∼3 ms, and it can evoke ∼0–800 Hz bandwidth frequency response. The observed temporal surface displacement dynamics include a baseline period before sample excitation representing the noise level, a primary deformation period driven by the air-pulse excitation force, and a subsequent period of damped oscillatory motion. The damping oscillation period in Figure 2B was obviously an under-damped conditional oscillation. The displacement dynamics *y*(*t*) in the damping oscillation period can be represented by Lan et al. (2020b)

$$y\left(t\right)=Ae^{-2\pi f_{n}\mathrm{\varepsilon t}}\sin\left[2\pi f_{n}\sqrt{1-\varepsilon^{2}}t+\varphi \right]$$ (2)

where *A* is the amplitude, and *φ* is a phase value. Eq. 2 consists of the product of an exponential function and a sinusoidal function, which respectively represent the amplitude decay trend and the oscillation frequency. In the sinusoidal function, the damping natural frequency $f_{d}=f_{n}\sqrt{1-\varepsilon^{2}}$ represents the dominant oscillation frequency, and it can be directly acquired by fast Fourier transform (FFT) (see the inset figure in Figure 2B). When the damping ratio *ε* is small (e.g., <0.3), the natural frequency *f*_*n*_ is in the range of 50–1,000 Hz, and the damped natural frequency *f*_*d*_ is nearly equal to the undamped natural frequency *f*_*n*_ (i.e., <3 Hz) (Lan et al., 2020b). Here, we assumed *f*_*d*_ = *f*_*n*_. Therefore, Eq. 2 can be simplified to

$$y\left(t\right)=Ae^{-2\pi f_{n}\mathrm{\varepsilon t}}\sin\left[2\pi f_{n}t+\varphi \right]$$ (3)

In the inset figure of Figure 2B, multiple frequency components were observed in the range of approximately 200–600 Hz, and the dominant oscillation frequency was 386 Hz, which was then substituted into Eq. 3 as *f*_*n*_ to fit the oscillation dynamics. In the fitting results, *A* = 0.358 μm, *ε* = 0.033. The *R* ^2^ of the fitting was 0.845, and the root mean squared error (RMSE) was 0.045 μm. The residual mismatch between the original displacement data and the fitting data occurred because SDOF was a simplified model that only considered the dominant frequency and discarded other frequency components. The variation of phase (*φ*) in time could also contributed to the residual error. The residual error could be further reduced if additional frequency components were used to describe the damping oscillation data using a multi degree of freedom (MDOF) method. It was noted that environmental vibrations can lead to low-frequency phase noise (e.g., ∼10–40 Hz) (Lan et al., 2017). Therefore, a high-pass filter was applied to the temporal displacement curves to reduce vibrational phase noise prior to SDOF modeling.

### Point-to-Point M-Mode Measurement

OCE measurements were performed using a point-to-point M-mode measurement method with a fixed distance (3 mm) between the stimulation and measurement points at sample surface. For each measurement point, a repeated A-scan (M-mode) was performed to acquire the phase variation over time and convert the phase change into the induced displacement profiles using Eq. 1. The samples were moved using a translation stage to spatially measure the resulting tissue dynamics. Because the dominant natural frequency was not dependent upon the stimulation force magnitude, as demonstrated in our previous work with agar phantoms (Lan et al., 2020b) and *in vivo* corneas (Lan et al., 2021a). We would expect this holds for other samples, e.g., silicone, silicone-agar mixture phantoms, or the beef shank samples. The choice of air-pulse pressure can be more flexible on tissues with different stiffness. We adjusted the stimulation pressure over the range of 60–85 Pa to achieve obvious oscillation features (similar to Figure 2B), where the induced maximum negative displacements were generally in the range of 0.5–1.5 μm, and the maximum oscillation magnitude (*A* in Eq. 3) was in the range of 0.1–0.4 μm.

## Results

**FIGURE 3:**
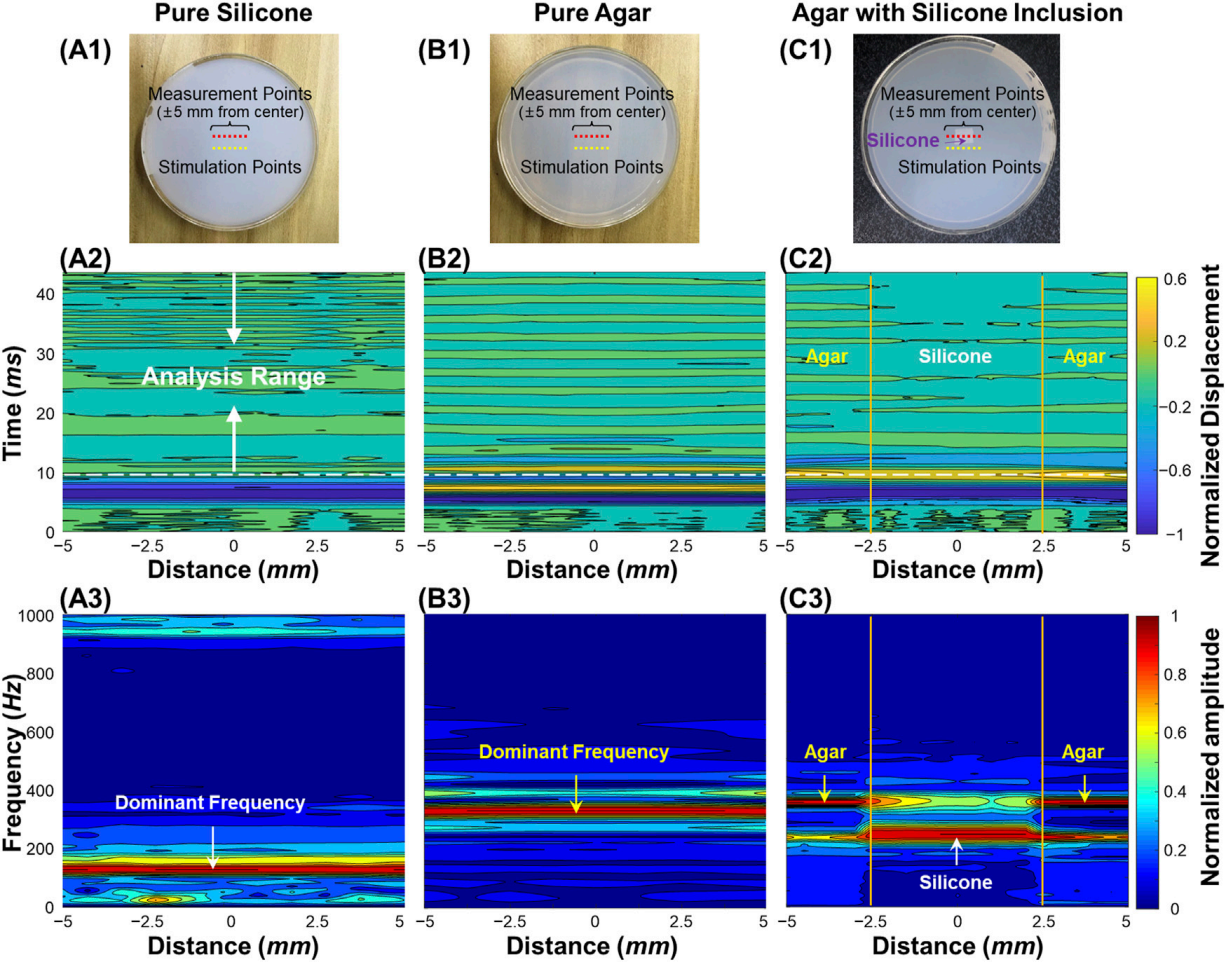
Oscillation feature analysis for the homogeneous and heterogeneous phantom samples. **(A1–C1)**: Top views of the silicone, agar, and silicone-agar mixture phantoms. The measurements were performed at 21 points to cover a 10 mm scan length. The distance between each stimulation and measurement point was 3 mm. **(A2–C2)**: Normalized surface displacement profiles in time domain. Fast-Fourier transformation was performed in the analysis range of 10–43 ms for oscillation frequency feature analysis. **(A3–C3)**: Normalized frequency spectrum in response to the measurement locations for different phantoms. The dominant oscillation frequencies were 128.7 ± 1.0 Hz for the silicone phantom (A3) and 329.7 ± 16.5 Hz for the agar phantom (B3). In (C3), the silicone block can be distinguished in the silicone-agar mixture phantom due to distinct frequency features, which were 247.9 ± 0.7 Hz for the silicone block and 357.6 ± 0.8 Hz for the agar basis.

Figure 3 demonstrates the air-pulse induced oscillation features in the time and frequency domains for the silicone phantom, the agar phantom, and the silicone-agar mixture phantom with a rectangular silicone block embedded in the center of the agar basis. Measurements were performed at a distance of 3 mm from each stimulation point (as shown in Figures 3A1–C1). OCE measurements were performed at 21 points to cover a 10 mm scan length. Figures 3A2–C2 demonstrate the normalized displacement profiles in the time domain for these phantoms. Across the measurement positions (±5 mm), the coefficients of variation (CVs, standard deviation/mean) of the measured displacements were 7.14% (silicone), 5.30% (agar), and 22.43% (silicone-agar mixture phantom). In Figure 3C2, the silicone region had relatively large negative displacements (−1.81 ± 0.04 μm) compared to the agar region (−1.53 ± 0.15 μm), indicating that the silicone was softer than the agar. This was consistent with the Young’s modulus measurements (silicone: 757.99 kPa; agar: 1,061.4 kPa). The oscillation periods (10–43 ms) of the displacement dynamics were analyzed using FFT, and the resulting frequency spectrums are shown in Figures 3A3–C3. The dominant oscillation frequencies were 128.7 ± 1.0 Hz for the silicone phantom (Figure 3A3) and 329.7 ± 16.5 Hz for the agar phantom (Figure 3B3). The differences in dominant oscillation frequencies indicated obvious differences in stiffness and elasticity (Young’s modulus) for these two phantoms as well. In the silicone-agar mixture phantom, the frequency components were more complex (Figure 3C3). There were two main oscillation frequency components across the measurement positions, and the magnitudes of these two frequencies varied in measurement positions and had similar but distinguishable values in the adjunction area. In the agar area, the dominant oscillation frequency was 357.6 ± 0.8 Hz; in the silicone block area, the dominant oscillation frequency was 247.9 ± 0.7 Hz. Notably, measurements of the embedded silicone block were affected by the surrounding 2% agar phantom, so that the silicone block in the silicone-agar mixture phantom had much higher (2 times) oscillation frequencies than the pure silicone phantom. When Eq. 2 (instead of Eq. 3) was used for natural frequency (*f*_*n*_) estimation, the damping ratios (*ε*) were small for the silicone (0.010 ± 0.004), agar (0.018 ± 0.006), and silicone-agar mixture phantoms (0.018 ± 0.009). The differences between *f*_*n*_ and *f*_*d*_ were less than 0.1 Hz, so *f*_*n*_ can be assumed to be equal to *f*_*d*_. The oscillation features reflected both the global and local properties for the silicone-agar mixture phantom. In each measurement position either in the silicone or the agar area, the sample oscillation dynamics included the oscillation frequency components for both the agar and silicone, indicating the global properties; the variation of the frequency magnitude over different areas indicated the local properties.

**FIGURE 4:**
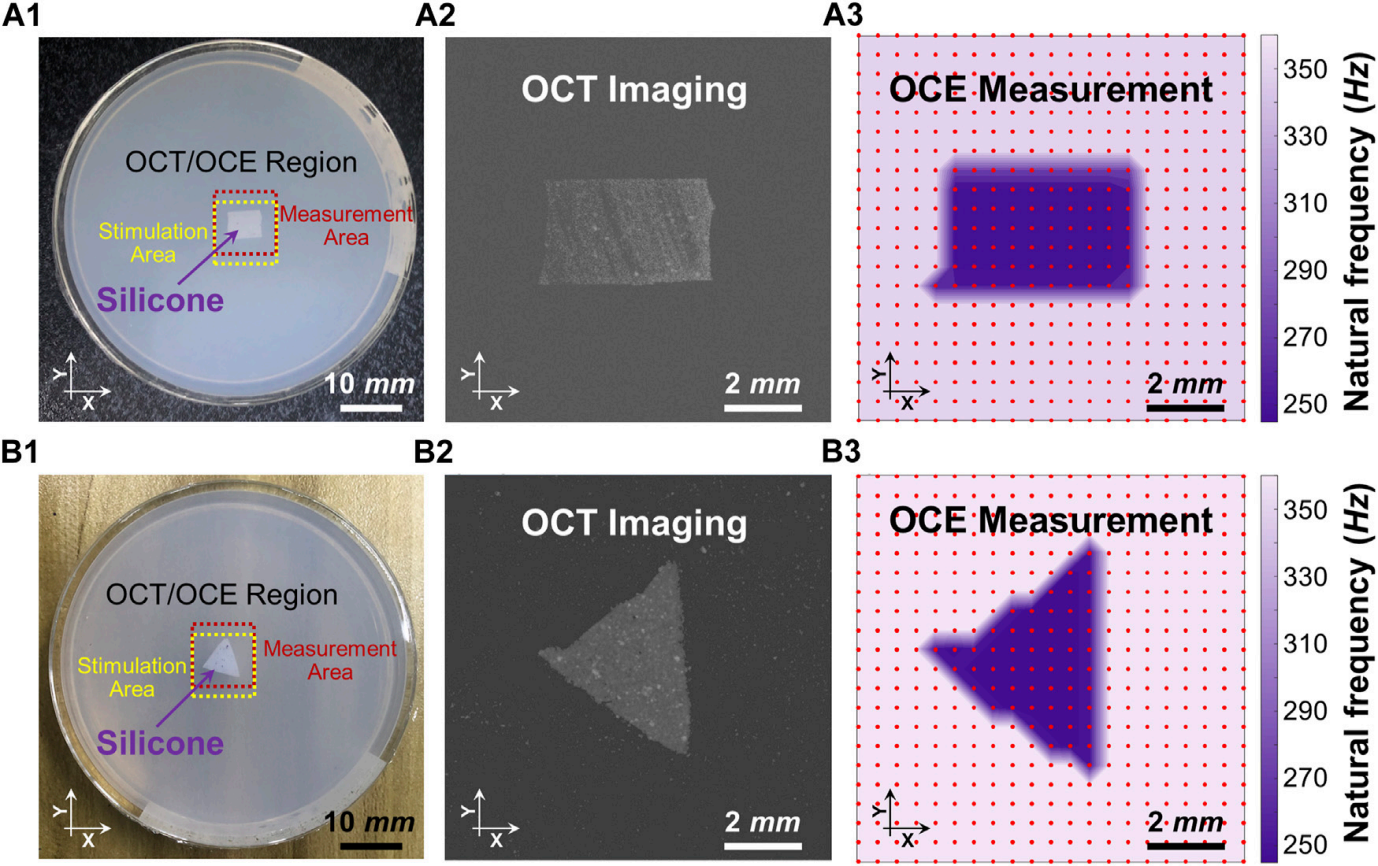
Distinguishing the embedded silicone blocks from the 2% agar using the spatialized dominant natural frequency measurement (*f*_*n*_). Panels **(A1)** and **(B1)** show the top views of the silicone-agar mixture phantoms and the positions of the stimulation and measurement. Panels **(A2)** and **(B2)** are the *en face* OCT images of the rectangular and triangular silicone blocks, respectively. Panels (A3) and (B3) shows spatial distribution of the dominant natural frequencies (*f*_*n*_) using the single degree of freedom method. In Panel **(A3)**, the dominant natural frequencies (mean ± SD) were 255.1 ± 11.0 Hz for the silicone block and 355.5 ± 1.4 Hz for the agar basis. In Panel **(B3)**, the dominant natural frequencies (mean ± SD) were 249.0 ± 4.6 Hz for the silicone block and 361.3 ± 5.5 Hz for the agar basis. Optical coherence tomography (OCT); optical coherence elastography (OCE).

Figure 4 shows the spatial distributions of the dominant natural frequency (*f*_*n*_) in the heterogeneous silicone-agar mixture phantoms, where a rectangular or triangular silicone block was embedded in 2% agar (Figures 4A1, 4B1). The air cannula and OCT beam were fixed with a distance of 3 mm in the *Y* direction, and the samples were moved using a translation stage to cover a 10 mm × 10 mm^2^ field of view using 21 × 21 sampling points. The sampling length was 0.5 mm in the *X* and *Y* directions. In Figure 4A3, the dominant natural frequencies (mean ± SD) were 255.1 ± 11.0 Hz for the silicone block and 355.5 ± 1.4 Hz for the agar basis. Similarly, in Figure 4B3, the dominant natural frequencies (mean ± SD) were 249.0 ± 4.6 Hz for the silicone block and 361.3 ± 5.5 Hz for the agar basis. The silicone blocks were clearly distinguishable from the agar basis using the natural frequency features. The OCE dominant natural frequency measurement can match the OCT imaging (Figures 4A2, B2).

**FIGURE 5:**
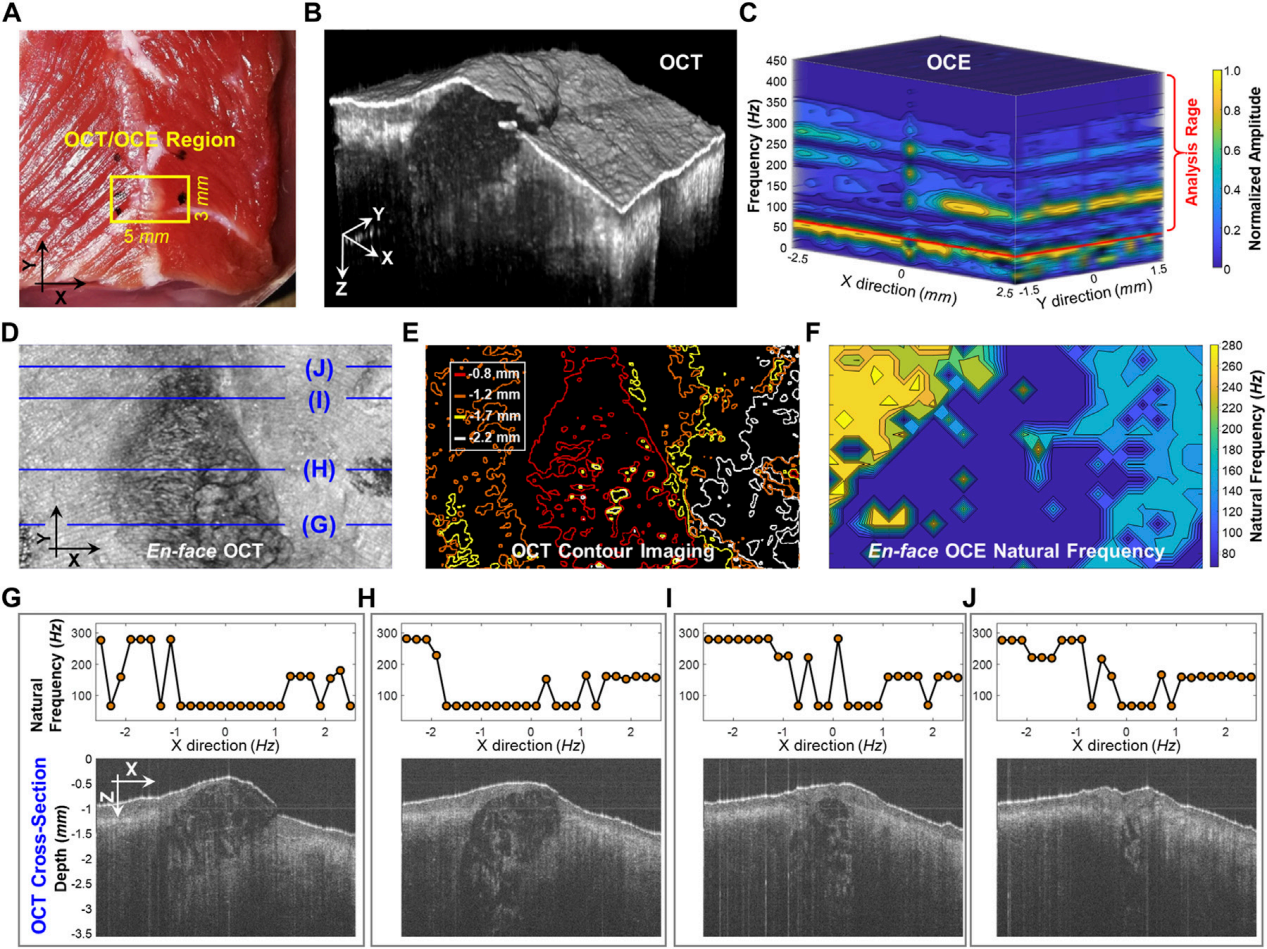
Spatial assessment of the dominant natural frequency (*f*_*n*_) distribution in a beef shank sample. **(A)** Top view photo of the beef shank sample. The boundary of the beef shank sample was sealed using 2% agar in a Petri dish with an inner diameter of 38 mm and a height of 13 mm. The small window shows the regions (5 × 3 mm) for both the optical coherence tomography (OCT) structural imaging and the optical coherence elastography (OCE) natural frequency measurement. **(B)** OCT volume scan for the beef tissue sample. **(C)** Normalized oscillation frequency spectrum in the *X* and *Y* directions. The *f*_*n*_ for each measurement position was then estimated in the range of 70–450 Hz. **(D)** and **(E)** show the *en face* OCT imaging and the corresponding contour maps at different depths (0.8, 1.2, 1.7, and 2.2 mm). **(F)** Spatial distribution of natural frequency (*f*_*n*_). **(G–J)** Comparison between the *f*_*n*_ values and the OCT cross-sectional structures at the X-Z planes registered in **(D)**.

Figure 5 compares the structural imaging and OCE measurement of the dominant natural frequency (*f*_*n*_) for the beef shank sample. The OCT/OCE measurement region (marked by ink points) was 5 × 3 mm, which included a mixture of connective tissues (e.g., fat, fascia) centered between muscle fibers on both sides (see top view in Figure 5A). Figure 5B and Figure 5D respectively show the OCT volume scan and the *en face* image for the beef sample. Figure 5E shows the depth-dependent contour maps of the tissue structure at the depths of 0.8, 1.2, 1.7, and 2.2 mm. The contour maps were acquired using the MATLAB language (R2019b, MathWorks, Inc.). We used the median filter (neighborhood size: 5 pixels × 5 pixels) to smooth the 8-bit *en face* imaging at each depth, and binarized the image with an intensity threshold of 120, and then detected the image edges using the Sobel operator. The OCE sampling was 26 points × 16 points (X × Y) with the spatial sampling of 0.2 mm in both directions. Figure 5C shows the normalized oscillation frequency spectrum in both the *X* and *Y* directions for the beef tissue sample. Notably, each position had similar low frequency components (e.g., <70 Hz) but different high frequency components (e.g., 70–450 Hz). We set a window of 70–450 Hz to estimate the dominant natural frequency (*f*_*n*_) using the SDOF method (Eq. 3). Figure 5F shows the spatial distribution of the dominant natural frequency (*f*_*n*_) in the *X* and *Y* directions. Comparing the *en face* image, depth-dependent contour maps, and the natural frequency distributions in Figures 5D–F, we noticed that the natural frequency values correlated with tissue structure and compositions in both the lateral (X and Y) and axial/depth (Z) directions. Figures 5G–J compare *f*_*n*_ and the corresponding OCT cross-sections. The central connective tissue had obvious lower natural frequencies (91.7 ± 58.2 Hz) than the muscle regions. The muscle region on the left side had higher frequency (252.6 ± 52.3 Hz) than the muscle region on the right side (161.5 ± 35.8 Hz) due to the sample thickness difference. Using the SDOF method, we can produce a general depiction of the material difference (connective tissue and muscle) and structural difference (e.g., shape and thickness) based on the spatial OCE measurements of the dominant natural frequencies. However, the method does not have sufficient spatial resolution to precisely distinguish the boundaries between fat and muscle or to discern muscle fibers and their orientations.

## Discussion

In this study, we spatially characterized the dominant natural frequency distributions for heterogeneous tissues and samples using the low-force OCE method, and we demonstrated that the natural frequency property is not only a global factor determined by mass, stiffness, and thickness (Crecea et al., 2009; Qi et al., 2013; Lan et al., 2020b; Lan et al., 2021a), but it is also affected by local features, such as regional variations in shape, stiffness, and material. In the frequency spectrum after the FFT process of the oscillation period, multiple frequency components were usually observed, and these components reflected the global properties. As the magnitude of each frequency component varied across the sample, the dominant natural frequency *f*_*n*_ for a specific measurement position could represent the dominant feature in that region. Since the resonant frequency features are a product of the material properties (e.g., Young’s moduli) as well as many other factors, the measured natural frequency of the same material can be very different as the mass, morphology or boundary conditions are different. In this study, we measured the distribution of natural frequencies to spatially distinguish the relative differences in tissue stiffness, structure and composition within heterogeneous tissues and tissue mimicking phantoms. This natural frequency OCE approach could provide a clinically viable, quantitative method to detect local tissue stiffness variations in ocular tissues induced by injuries, pathological degenerations, surgical, or medical treatments.

In a well-controlled phantom with a silicone block embedded in agar to mimic tissue lesions surrounded by normal tissue (Figure 3C1), the two materials had measurably different Young’s moduli (silicone: 757.99 kPa; agar: 1,061.40 kPa) and clear boundaries. We observed different oscillation patterns in the silicone and agar regions (Figure 3C2) as well as distinct frequency features in the oscillation frequency spectrum (Figure 3C3). An ideal two-peak frequency spectrum appeared at each of the measurement positions across the agar/silicone/agar regions, indicating that the oscillation frequency components can reflect the global properties of the phantom. We also observed that the magnitude of each peak frequency changed as the measurement location changed. The frequency with the maximum magnitude was 357.6 ± 0.8 Hz in the agar region and 247.9 ± 0.7 Hz in the silicone region. When measurement positions were nearer to the silicone inclusion, we observed lower oscillation frequencies. Thus, the local sample properties are detectable from the oscillation frequency spectrum (as shown in Figure 3C3). Thereby, we can conclude that the measurement of the oscillation frequency components can reflect both the global and local features of the sample. In the resonant frequency spectrum, the numbers of the frequencies may relate to the global properties, whereas the variation of the magnitude of each frequency component at each measurement position may relate to the local properties. To further test our hypothesis, we used the SDOF method to calculate the dominant natural frequency (*f*_*n*_) for each measurement position and access the spatial distribution of the dominant *f*_*n*_ in the heterogeneous phantoms (Figure 4). Both the rectangular and triangular silicone blocks were clearly distinguishable from the agar basis using natural frequency features, and the OCE dominant natural frequency plot matched the OCT imaging well. In the ideal tissue-mimicking phantoms, we can clearly distinguish tissue boundaries using the dominant natural frequencies via the SDOF method (Figure 4A3 and Figure 4B3). It should be noted that tissue natural frequency is not dependent upon elastic modulus alone, but is also determined by other factors, such as mass, thickness, shape, and boundary conditions. That is consistent with our observation that the embedded silicone block in the silicone-agar phantom had twice the natural frequency (247.9 ± 0.7 Hz; Figure 3C3) compared to the pure silicone phantom (128.7 ± 1.0 Hz; Figure 3A3).

In the spatial measurement of the beef shank sample (Figure 5), the frequency spectrum and the dominant natural frequency distribution became more complex than those in the ideal tissue-mimicking phantoms. Although we were able to roughly characterize the material difference (connective tissue and muscle) and the structural difference (e.g., shape and thickness) using the dominant natural frequency values, our method did not offer enough spatial resolution to totally distinguish the boundaries between connective tissue and muscle or to discern muscle fibers and their orientations. There are several possible reasons for the low spatial resolution in the natural frequency measurement. First, the measurement of natural frequency reflects both the local factor near the measurement point and the global factor in lateral and depth directions, while the beef sample was obviously heterogeneous in each direction. Therefore, tissue boundary may not be distinguished clearly based on the resonant features because of the effect of the structure and material adjacently and globally. Second, The SDOF method is only an approximation and simplification method that used the dominant frequency in the frequency spectrum (Figure 5C) while disregarding other frequency features which may reflect subtle structural or material variations. A better analytical model that considers multiple resonant frequency features may better interpret the resonant features and obtain the spatial variations in material and structure. The multi degree of freedom (MDOF) method can be used to describe a more complex system and the general vibration of the system consists of a sum of all the vibration modes, and each vibration mode vibrates at its own frequency. Developing a reliable MDOF method will be the direction of our future work for better characterization of tissue biomechanics. Third, the spatial sampling was only 0.2 mm, which may be insufficient to capture the local changes in tissue stiffness or structure, such as muscle fibers shown in Figure 5B and Figure 5D. Denser OCE sampling may provide better spatial resolution for natural frequency characterization but require more data acquisition time.

We are enthusiastic about the possibility of using natural frequency observations for clinical characterization of tissue stiffness. Nevertheless, the multi-modal combination of OCT imaging and natural frequency measurements is a complex and unsolved problem. While it is intriguing to consider the collapsed projection of an OCT image, the structural complexity of the underlying tissue cannot be fully represented using *en face* or B-mode projections. Similarly, the natural frequency projections shown in Figure 5 are an incomplete picture of the underlying mechanical complexity of the samples. Despite these shortcomings, these results are encouraging steps towards a strategy to link structural and mechanical tissue properties.

Additional work is required to adapt this natural frequency OCE method to clinically map corneal biomechanics. A major challenge is that the cornea has a complex tissue organization with a spatially inhomogeneous structure that is directionally anisotropic in its response to loading. Corneal collagen fibrils are preferentially oriented along the superior-inferior and nasal-temporal meridians in the central region and have circumferential orientation in the periphery (Meek, 2009; Chong and Dupps, 2021). The regional and directional uniformity of the collagen fibril organization causes stiffness differences of the cornea in the lateral dimension. Second, collagen fibrils exhibit greater interconnectivity in the anterior third of the corneal stroma than in the posterior stroma, resulting in a nonuniform strength through the depth dimension of the cornea (Komai and Ushiki, 1991). Third, the cornea is composed of many extracellular materials and components with variable charge and chemical interactivity that are distributed inhomogeneously throughout the cornea. For example, the more hydrophilic glycosaminoglycans are found in the deep stroma and promote nonuniform swelling and viscous behaviors (Chong and Dupps, 2021). In addition, ocular disease progression (e.g., keratoconus, ectasia, and glaucoma) and treatments (e.g., corneal cross-linking, refractive surgery) can also add complexity to the regional and directional variations in corneal structure and biomechanical behaviors. How to interpret the pathological or treatment outcomes in corneal biomechanical behaviors from subtle spatial changes in corneal oscillation frequency spectrum, or dominant frequency components, or the variation of magnitude for each main frequency component is challenging but will be an essential part of our research moving forward.

We will further investigate the correlation between corneal natural frequencies and other ocular structural parameters (such as corneal thickness, intraocular pressure, and corneal topography) to better understand the limitations and potential clinical use of the spatial natural frequency measurement method using the low-force OCE system. More advanced analytical methods and finite element eye models must be developed to consider the interactivities among parameters such as corneal biomechanics, intraocular pressure, corneal shape and micro- and macro-compositions, and boundary conditions. Developing such analytical models and investigating the relationships between corneal natural frequency and the above factors could help develop methods to better characterize corneal biomechanical properties. Future studies on patients with ocular disease (e.g., keratoconus or glaucoma) or myopic degeneration, or those who have undergone medical treatments or surgical interventions, are needed to understand the clinical utility of this method for the detection and classification of corneal abnormalities, and for the evaluation of treatment outcomes. Comparison between this OCE method and the clinical biomechanical parameters derived from the Ocular Response Analyzer and the CorVis ST on normal patients and patients with ocular disease or after treatments will also be useful.

## Data Availability

The original contributions presented in the study are included in the article/Supplementary Material, further inquiries can be directed to the corresponding authors.
